# Supplementary material for: Mycoplasma genitalium adhesin P110 binds sialic-acid human receptors
Source: Nat Commun. 2018 Oct 26;9:4471. doi: 10.1038/s41467-018-06963-y (PMC6203739; doi:10.1038/s41467-018-06963-y)
Supplement: Supplementary file 1 — Supplementary Information [file 41467_2018_6963_MOESM1_ESM.pdf]

# *Mycoplasma genitalium* adhesin P110 binds sialic-acid human receptors

**Aparicio et al**

**Supplementary Information**

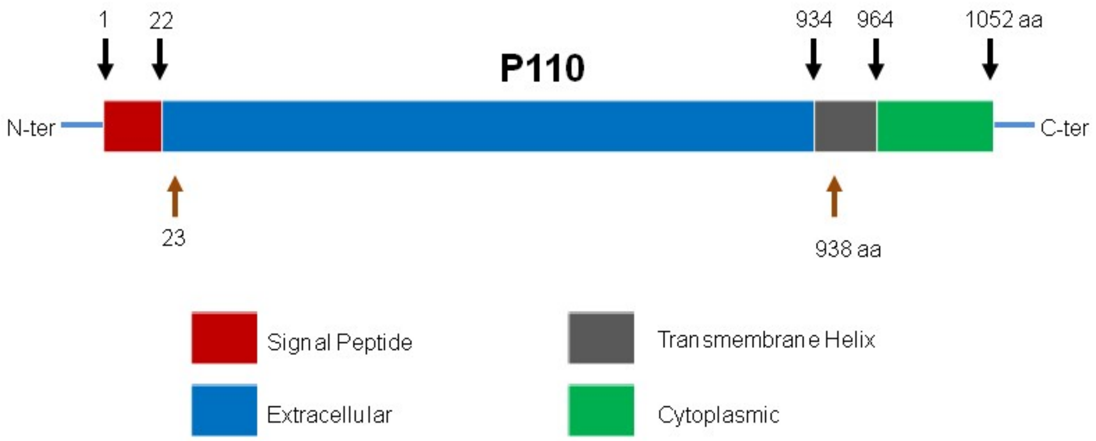


**Supplementary Figure 1. Protein domains of P110 and construct (erP110) designed for this study.** Protein domains predicted by Psi-Pred server[1] are showed in red, blue, gray and green for the signal peptide, extracellular, transmembrane and cytoplasmic domains respectively. The signal peptide has also been revised with computational program SignalIP 4.1 with a cutoff of 0.34[2]. Brown arrows depict the erP110 construct.

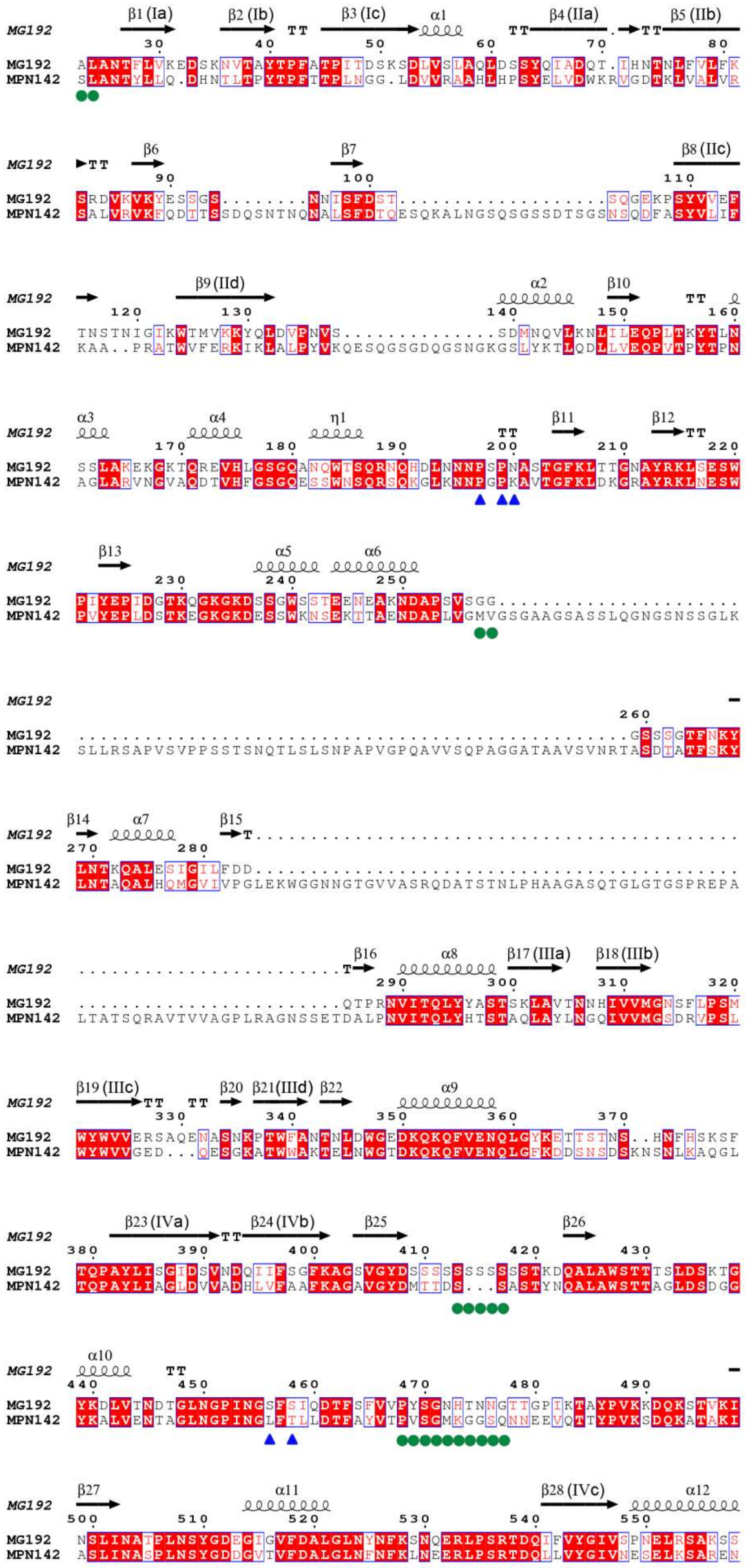

MG192
MPN142
β1 (Ia)
β2 (Ib)
β3 (Ic)
α1
β4 (IIa)
β5 (IIb)
β6
β7
β8 (IIc)
β9 (IId)
α2
β10
α3
α4
η1
β11
β12
β13
α5
α6
β14
α7
β15
β16
α8
β17 (IIIa)
β18 (IIIb)
β19 (IIIc)
β20
β21 (IIId)
β22
α9
β23 (IVa)
β24 (IVb)
β25
β26
α10
β27
α11
β28 (IVc)
α12

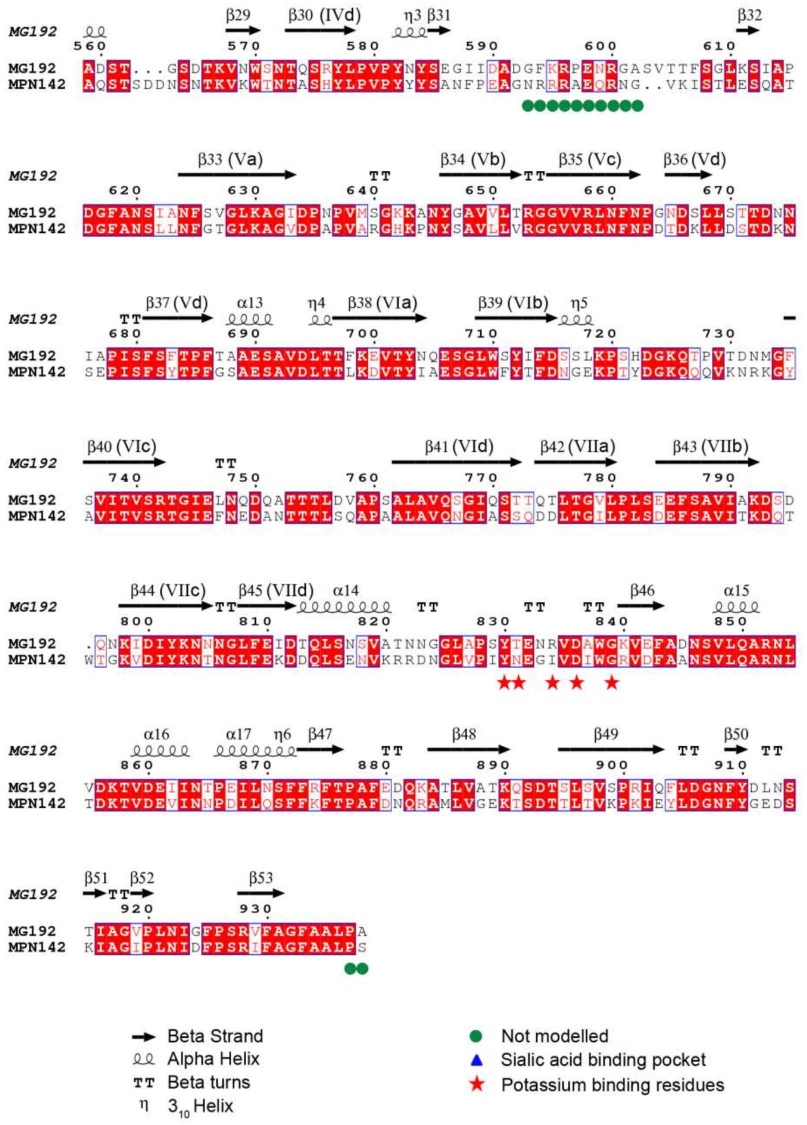

**Supplementary Figure 2. *M. genitalium MG192* and *M. pneumoniae MPN142* sequence alignment.** Protein alignment with secondary structure predicted by DSSP[3] and visualized by Espript [4]. Blades and β-Strands forming the β-Propeller are indicated as I-VII and a-d, respectively. Residues corresponding to the binding pocket, the potassium binding site or missing in the P110 model are depicted as blue triangles, red stars and green circles, respectively. Secondary structure elements are also showed.

A

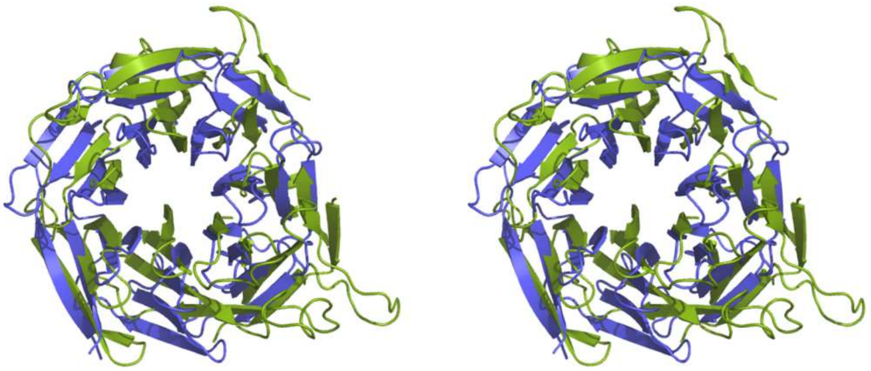

**Supplementary Figure 3. Wall-eyed stereo view of the superposition of erP110 onto Virginiamycin B Lyase.** The structural relationship was obtained by Dali Server network service. Superposition of the β-Propeller of P110 onto the regular β-Propeller of Virginiamycin B Lyase (PDB accession code 2Z2N) resulted in a r.m.s.d. of 3.6 Å.

A

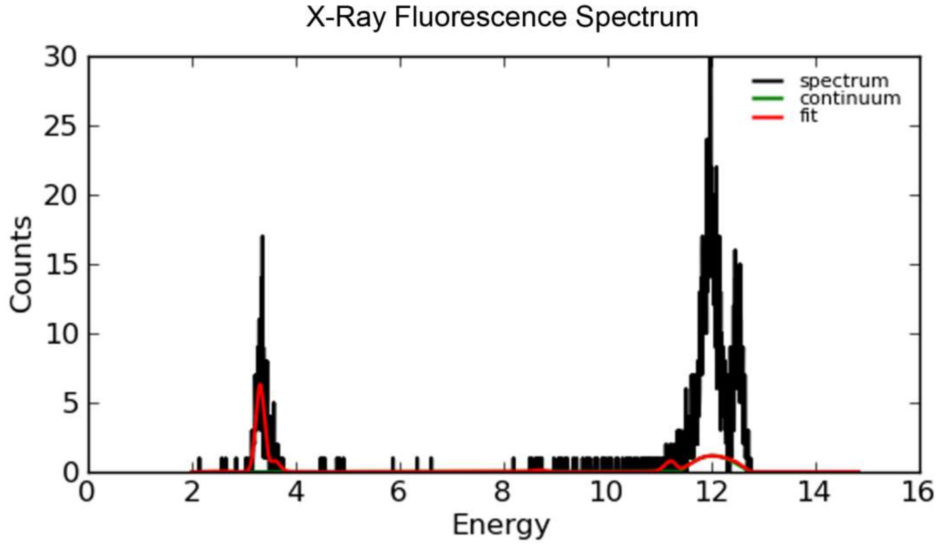


**Fit Peak Results:**

| Element | Group | Fit Area | Sigma | Energy | Ratio | FWHM | Chi square |
|---|---|---|---|---|---|---|---|
| S | K | 7.926440e-02 | 7.70e+00 | | | | |
| | KL3 | 7.465518e-02 | 7.25e+00 | 2.307 | 0.94185 | 0.180 | 0.01 |
| | KM3 | 4.609225e-03 | 4.48e-01 | 2.464 | 0.05815 | 0.182 | 0.02 |
| Cl | K | 2.761359e+00 | 7.94e+00 | | | | |
| | KL3 | 2.555113e+00 | 7.35e+00 | 2.622 | 0.92531 | 0.184 | 0.03 |
| | KM3 | 2.062459e-01 | 5.93e-01 | 2.816 | 0.07469 | 0.187 | 0.03 |
| K | K | 3.008673e+02 | 1.87e+01 | | | | |
| | KL3 | 2.696824e+02 | 1.68e+01 | 3.313 | 0.89635 | 0.193 | 0.94 |
| | KM3 | 3.118490e+01 | 1.94e+00 | 3.590 | 0.10365 | 0.196 | 0.84 |
| Ca | K | 7.311460e+00 | 9.11e+00 | | | | |
| | KL3 | 6.495281e+00 | 8.10e+00 | 3.690 | 0.88837 | 0.198 | 0.39 |
| | KM3 | 8.161782e-01 | 1.02e+00 | 4.013 | 0.11163 | 0.202 | 0.00 |
| Cr | K | 1.170350e-02 | 9.14e+00 | | | | |
| | KL3 | 1.036932e-02 | 8.10e+00 | 5.412 | 0.88600 | 0.218 | 0.00 |
| | KM3 | 1.334186e-03 | 1.04e+00 | 5.947 | 0.11400 | 0.224 | 0.01 |
| Mn | K | 1.531163e+00 | 9.35e+00 | | | | |
| | KL2 | 4.559192e-01 | 2.78e+00 | 5.888 | 0.29776 | 0.223 | 0.01 |
| | KL3 | 8.944445e-01 | 5.46e+00 | 5.899 | 0.58416 | 0.223 | 0.01 |
| | KM3 | 1.807998e-01 | 1.10e+00 | 6.490 | 0.11808 | 0.229 | 0.02 |
| Fe | K | 1.552949e+00 | 9.43e+00 | | | | |
| | KL2 | 4.627633e-01 | 2.81e+00 | 6.391 | 0.29799 | 0.228 | 0.02 |
| | KL3 | 9.062545e-01 | 5.50e+00 | 6.404 | 0.58357 | 0.228 | 0.02 |
| | KM3 | 1.839313e-01 | 1.12e+00 | 7.058 | 0.11844 | 0.235 | 0.00 |
| Co | K | 4.630978e-02 | 9.51e+00 | | | | |
| | KL2 | 1.380356e-02 | 2.83e+00 | 6.915 | 0.29807 | 0.234 | 0.00 |
| | KL3 | 2.699490e-02 | 5.54e+00 | 6.930 | 0.58292 | 0.234 | 0.00 |
| | KM3 | 5.511327e-03 | 1.13e+00 | 7.649 | 0.11901 | 0.241 | 0.00 |
| Ni | K | 1.710491e-01 | 9.60e+00 | | | | |
| | KL2 | 5.099316e-02 | 2.86e+00 | 7.461 | 0.29812 | 0.239 | 0.00 |
| | KL3 | 9.962926e-02 | 5.59e+00 | 7.478 | 0.58246 | 0.239 | 0.00 |
| | KM3 | 2.042668e-02 | 1.15e+00 | 8.265 | 0.11942 | 0.247 | 0.02 |
| Cu | K | 4.744064e-01 | 9.69e+00 | | | | |
| | KL2 | 1.419078e-01 | 2.90e+00 | 8.028 | 0.29913 | 0.245 | 0.01 |
| | KL3 | 2.764955e-01 | 5.65e+00 | 8.048 | 0.58282 | 0.245 | 0.01 |
| | KM3 | 5.600311e-02 | 1.14e+00 | 8.905 | 0.11805 | 0.253 | 0.08 |
| Zn | K | 9.511501e+00 | 9.87e+00 | | | | |
| | KL2 | 2.839754e+00 | 2.95e+00 | 8.616 | 0.29856 | 0.251 | 0.05 |
| | KL3 | 5.526372e+00 | 5.74e+00 | 8.639 | 0.58102 | 0.251 | 0.05 |
| | KM3 | 1.145375e+00 | 1.19e+00 | 9.572 | 0.12042 | 0.260 | 0.08 |
| Se | K | 5.712985e+01 | 1.31e+01 | | | | |
| | KL2 | 1.679046e+01 | 3.86e+00 | 11.182 | 0.29390 | 0.274 | 0.45 |
| | KL3 | 3.253431e+01 | 7.48e+00 | 11.222 | 0.56948 | 0.275 | 0.51 |
| | KM3 | 7.398887e+00 | 1.70e+00 | 12.494 | 0.12951 | 0.286 | 4.09 |
| | KN3 | 4.061932e-01 | 9.34e-02 | 12.652 | 0.00711 | 0.287 | 2.86 |

**Supplementary Figure 4. X-ray Fluorescence data of P110 crystals.** Potassium ion found in the crystal structure of P110 was confirmed by X-ray fluorescence. A) The energy peak observed in the spectrum fits well with the expected energy for a potassium ion. Blue arrow and rectangle shows the K fitting statistics.

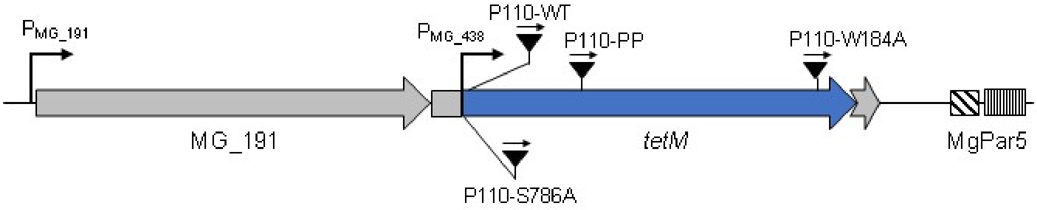


**Supplementary Figure 5. Insertion site of the TnPacP110 minitransposon.** Chromosome showing the mutants selected for cytadherence analysis. Black triangles denote the insertion site and orientation of the different TnPacP110 minitransposons within the *tetM* marker.

**Data collection and Refinement Statistics [a]**

| | SeMet | Native | 3'-Sialyllactose | 6'-Sialyllactose |
|---|---|---|---|---|
| **Data collection** | | | | |
| **Space group** | I222 | I222 | I222 | I222 |
| **Cell dimensions** | | | | |
| **a,b,c (Å)** | 109.93, 152.91, 174.69 | 109.60, 152.94, 172.71 | 110.03, 151.57, 176.55 | 109.69, 152.69, 176.23 |
| **α,β,γ (º)** | 90.00, 90.00, 90.00 | 90.00, 90.00, 90.00 | 90.00, 90.00, 90.00 | 90.00, 90.00, 90.00 |
| **Unique reflections** | 33663 (2469) | 39641 (2903) | 73757 (10661) | 50406 (3692) |
| **Resolution (Å)** | 59.07-2.95 (3.04-2.95) | 62.01-2.70 (2.77-2.73) | 115.00-2.21 (2.33-2.21) | 57.70-2.50 (2.56-2.50) |
| **Wavelength (Å)** | 0.9791 | 1.1271 | 0.9789 | 0.9789 |
| **Rmeas (%)[b]** | 0.11 (1.16) | 0.05 (0.83) | 0.05 (1.02) | 0.04 (0.72) |
| **I/σI** | 15.23 (1.90) | 19.1 (1.8) | 20.06 (1.70) | 18.90 (1.90) |
| **Completeness (%)** | 99.90 (99.90) | 98.90 (98.70) | 99.80 (99.80) | 98.40 (98.10) |
| **Redundancy** | 13.01 (11.00) | 4.60 (4.80) | 4.80 (4.90) | 4.30 (4.50) |
| **<d’’/sig>** | 3.07 (0.81) | | | |
| **B-Model Refinement statistics** | | | | |
| **Resolution** | | 58.95-2.73 (2.73) | 115.00-2.21 (2.21) | 57.70-2.52 (2.52) |
| **Num. of reflections** | | 36543 | 70044 | 46747 |
| **$R_{cryst}$ (%)[c]** | | 18.45 | 18.92 | 18.89 |
| **$R_{free}$ (%)[d]** | | 22.92 | 22.13 | 23.29 |
| **No. residues** | | 911 | 911 | 911 |
| **No. ligands** | | 0 | 1 | 1 |
| **Solvent content (%)** | | 66 | 66 | 66 |
| **Av. B-factor ($Å^2$)** | | 96 | 76 | 98 |
| **Coor. Error (Å)[e]** | | 0.26 | 0.16 | 0.24 |
| **Rms dev.bonds (Å)** | | 0.02 | 0.03 | 0.03 |

| | | | |
|---|---|---|---|
| **Rms dev.angles (°)** | 2.1 | 2.4 | 2.4 |

**[a] Values in parentheses correspond to the highest resolution shell.**

**[b] $R_{sym} = \Sigma_{hkl}\Sigma_i|I_i(hkl) - <I(hkl)>| / \Sigma_{hkl}\Sigma_i I_i (hkl)$, where $I_i(hkl)$ is the intensity of an observation and $<I(hkl)>$ is the mean value of observations for a unique reflection.**

**[c] $R_{cryst} = \Sigma_h|Fo(h) – Fc(h)| / \Sigma_h|Fo(h)$, where Fo and Fc are the observed and calculated structure-factor amplitudes, respectively.**

**[d] $R_{free}$ was calculated with 5% of data, which was excluded from the refinement.**

**[e] Based on maximum likelihood.**

**Supplementary Table 1. X-ray data collection and refinement statistics.**

| Primer name | Sequence (5’→3’) |
|---|---|
| Pac-F | GCGGAATTCTAGTATTTAGAATTAATAAAGTATGACTGAATATAAAC CTACTG |
| Pac-R | GCGGGATCCCTCGAGAATCTATGTCGACTTAAGCACCAGGTTTTCTA G |
| COMmg192-F | AGTGGGCCCACTAACAAAAAACAAATTAGTGATGTTGTTAGTGATTG TGTGAAAAAAATTGTTTATAATTAAGTTTGTATGAAAACAATGAGAA AACAG |
| COMmg192-R | AGTCTCGAGCTAACTTTTGGTTTCTTCTG |
| S783Amg192-F | GAGTACTCCCACTTGCCGAGGAATTCAGTG |
| S783Amg192-R | CACTGAATTCCTCGGCAAGTGGGAGTACTC |
| W184Amg192-F | GGCAAGCAAATCAGGCCACCAGTCAACGCA |
| W184Amg192-R | TGCGTTGACTGGTGGCCTGATTTGCTTGCC |
| PPmg192-F | CCTAAACAACAATGCCAGTGCCAATGCTTCAACTG |
| PPmg192-R | CAGTTGAAGCATTGGCACTGGCATTGTTGTTTAGG |
| S458Dmg192-F | TCAATGGGAGTTTTGACATCCAAGACACCT |
| S458Dmg192-R | AGGTGTCTTGGATGTCAAAACTCCCATTGA |
| Loopmg192-F | ATCAATGGGAGTTTTTTCAGCTTTGTTGTTCCTTATTC |
| Loopmg192-R | AACAACAAAGCTGAAAAAACTCCCATTGATCGGAC |
| Tnp3 | GATTCATGATTATATCGATCAAC |
| RTPCRmg192-R | CAGGGGCAATTGATTTAAGC |
| RTPCRmg192-F | TCCCCTAATGAATTGCGAAG |
| PacUp | GTAGCTAATCTAACAGTAGG |
| PacDw | GTCCTAGAACTTGGTGTATG |
| P110F | AGGAGATATACCATGGCACTGGCAAATACCTTTC |
| P110R | GTGATGGTGATGTTTAGGCAGTGCTGCAAAAC |

**Supplementary Table 2. Primers used in this study.**

| Strain | Description | Source |
|---|---|---|
| G37 | Wild-type | ATCC 33530 |
| ΔMG_192 | Deletion of the MG_192 gene by allelic exchange | Burgos *et al.*, 2006 |
| P110-WT | Re-introduction of a MG_192 wild-type allele in a ΔMG_192 mutant | This work |
| P110-S786A | Introduction of a MG_192 allele bearing a S783A substitution in a ΔMG_192 mutant | This work |
| P110-W184A | Introduction of a MG_192 allele bearing a W184A substitution in a ΔMG_192 mutant | This work |
| P110-PP | Introduction of a MG_192 allele bearing a P197A and P199A substitutions in a ΔMG_192 mutant | This work |
| P110-S458D | Introduction of a MG_192 allele bearing a S458A substitution in a ΔMG_192 mutant | This work |
| P110-Δloop | Introduction of a MG_192 allele bearing a S458-T462 deletion in a ΔMG_192 mutant | This work |

**Supplementary Table 3. Strains used in this study.**

## Supplementary References


1. Jones, D.T. Protein secondary structure prediction based on position-specific scoring matrices. *J Mol Biol* **292**, 195-202 (1999).
2. Nielsen, H., Engelbrecht, J., Brunak, S. & von Heijne, G. Identification of prokaryotic and eukaryotic signal peptides and prediction of their cleavage sites. *Protein Eng* **10**, 1-6 (1997).
3. Joosten, R.P. et al. A series of PDB related databases for everyday needs. *Nucleic Acids Res* **39**, D411-9 (2011).
4. Robert, X. & Gouet, P. Deciphering key features in protein structures with the new ENDscript server. *Nucleic Acids Res* **42**, W320-4 (2014).
